# The cardio-protective diet

**Published:** 2010-11

**Authors:** S. Sivasankaran

**Affiliations:** *Department of Cardiology, Sree Chitra Tirunal Institute for Medical Sciences & Technology, Thiruvananthapuram, Kerala, India*

**Keywords:** Adiposopathy, beta cell protection, cardiovascular diseases, eating behavior, prudent diet, sarcopenic adiposity

## Abstract

Globalization has made calorie rich, cheap, convenient marketed foods the main menu for the common man. Indians are particularly susceptible to the adverse outcomes of this dietary change because of ethnic, epigenetic reasons and sarcopenic adiposity (less muscle more fat for the same body weight). Children have smaller body frame making them more susceptible to adverse effects of hyperglycaemia leading to stress on beta cells and their damage. This has resulted in escalation of lifestyle diseases by three-fold, that too at our younger age group at lower body mass indices. Preventive measures are necessary in early life to protect the beta cells, to achieve a metabolically healthy society. This will help in sustaining optimal beta cell function throughout a person’s life. Modification in dietary habits by educating the society, proper food labelling and legal regulation, restricting calorie, sugar, saturated fat, trans-fat and salt intake has proved its benefits in the developed world. Changes in the quality of food is as important as restricting calorie intake. This includes facilitation of increased consumption of dietary fiber, complex carbohydrates, nuts, fruits and vegetables. Restrictions are needed to reduce trans-fats, saturated fats and cooking habits such as deep frying which oxidizes cholesterol and lipids. Foods with long shelf-life shorten the life line because of their salt, sugar or trans-fat content. Individual meals need to be targeted in the general dietary guidelines, to minimize the post-prandial metabolic insult. In general, we need healthy start to early life particularly the first twenty years of life so that the habits cultured during childhood are sustained for the rest of productive years.

## Introduction

The ideal cardio-protective diet remained elusive for decades because of the wide variation in the availability of food and cultural practices^1^,^2^. But the science of nutrition has given clear guidelines to achieve substantial control of the cardiovascular epidemic^3^. The present article outlines the basic strategies for providing a cardio-protective diet based on available evidence so as to get a clear idea regarding the dietary modification for the common man to achieve optimal heart health in India by 2020. A new paradigm is suggested to target individual meals to protect the insulin secreting beta cells of the pancreas, and reduce the inflammation of fat cells, the ‘adiposopathy’, in order to delay the early onset of risk factors, diabetes and cardiovascular disease in Indians^4^–^7^.

## Does dietary prevention work?

All developed nations have documented a substantial decline of the order of 50 per cent in the morbidity and mortality due to cardiovascular disease in the last 3 decades by public health interventions^8^. The best examples are that of Finland, United States, United Kingdom and New Zealand^9^. Population wide strategy complimented by high risk strategy was the method adopted and validated^10^. The methods adopted were simple like reducing salt, sugar, saturated fat and trans-fats intake, in addition to improving physical activity with systematic efforts to reduce the use of tobacco. Schools in United States, United Kingdom and Australia have banned sales of crisps, chocolates and sugar sweetened beverages. Trans-fats (refers to the trans isomeric form of polyunsaturated fats which unlike the cis forms cannot be used by the body enzymes which have a shape specificity to the cis isomeric forms) have been reduced/banned in several parts of US and other countries which account for an annual reduction of 50,000 cardiovascular disease (CVD) deaths alone in United States^11^.

## Diet and CVD: The evidence

Best evidence for the diet heart hypothesis were derived from the longevity of Inuit’s of Greenland, Crete island in the Mediterranean belt and the Okinawans in Japan^2^,^12^,^13^. Taking the lead from the initial studies excellent dietary guidelines and population strategies were evolved over the last 40 years^3^,^12^,^14^. Good public education, simultaneous efforts at other lifestyle issues like improving the physical activity and reduction in tobacco use, accounted for this success. This resulted in more than 50 per cent reduction in cardiovascular mortality in the developed world^8^,^9^. Co-operation with the food Industry, food labelling, provision of alternatives, and simultaneous public health legislations were the key elements (Table). The efforts were to reduce the consumption of refined sugar, salt, saturated fats, trans-fats and to increase the consumption of dietary fiber, complex carbohydrates, fruits and vegetables^15^–^27^. The evolution of the modern diet can be traced back from the primate evolution when the primate biology lost the ability to synthesize vitamin C consequent to the good consumption of fruits^28^. But over the last ten-thousand years, the agricultural revolution brought cereals as major dietary source of energy because of its longer shelf life^29^. Dairy farming and poultry were added to the human lifestyle some 3000 years back. Over the last 150 years, salting, refining, hydrogenation and frying were added as additional methods of improving the taste and prolonging the shelf life of food substances. Refined sugar with added fat increased the energy density of the food materials by 6 times^30^. These dietary changes generated a major mismatch between the genetic make up of man and what he could metabolize^31^. The harmful components of the westernized diet reside in its energy density and its ability to have a long shelf-life^32^. In short, food items with longer shelf-life confer a shorter life span for the consumer. Converting the marketed food into regular menu by the urban poor because of the cheapness, long shelf-life and convenience has created a wildfire of lifestyle related diseases in the developing world^33^. Western diets are characterized by the frequent consumption of refined cereals, sugar, salt, egg, full fat dairy products, partially hydrogenated oils, red meat and fried items. In contrast, a prudent traditional diet stands ahead in the multitude of dietary patterns studied for cardio-protection^2^,^12^,^13^,^34^,^35^.

**Table:** Major interventions implemented as a policy to achieve a cardioprotective diet

Intervention	Country	Ref.	National policy
1. Reduction in salt intake	Australia	16	‘Pick the tick’ healthy food sign.
2. Reduction in saturated fat	Mauritius	27	Substitution of soybean oil instead of palm oil in the public distribution
3. Reduction in animal fat, dairy products, and salt and increasing fruit and vegetable consumption	Finland	23	Mass education, campaign to change over to alternatives like berry cultivation from animal farming
4. Banning junk food at school, *e.g.*, crisps, chocolates and sugary drinks	USA, UK and Australia	24,25	Mass education, co-operation of the food industry, and legal actions
5. Minimizing trans-fats in commercial products	USA	11	Legal binding on food industry and local outlets to minimize trans-fats

## Diet and CVD: The Indian scenario

While the West observed a decline in CVD rate in the latter part of the 20^th^ century, India during the same period witnessed a 3-fold increase in the prevalence of cardiovascular diseases, risk factors and diabetes at a younger age, and lower body mass indices^36^–^39^. This increase has been largely attributed to rapid westernization. Rapid westernization is correlated in these studies with 2- to 5-fold increase in consumption of sugar, salt, high fat dairy products, eggs, red meat and oils with their trans-fat content^40^–^48^. Ghee-based sweets which contain oxidized cholesterol and hydrogenated fats is another contributing factor^49^–^51^. Vegetarianism prevalent in India is aptly criticized as the contaminated vegetarianism, consuming large amounts of fried foods, excess of salt, sugar and ghee^47^. Since cholesterol can be synthesized in the body, all recommendations advice to restrict dietary cholesterol consumption to be less than 300 mg/day, which is the average amount found in an ordinary egg yolk^48^. In India children are fed with double omelets which could contain double the amount of recommended daily intake of cholesterol that too in the oxidized form. Fresh fruits and vegetable, the protective components in the vegetarian diet are considerably lacking in Indian diet^32^,^52^. A mass movement is needed to re-culture today’s children to habituate to traditional diet, rich in fresh fruits and vegetables. Alcohol in Indian context has not shown any cardio-protective effect^40^.

## What makes Indians more susceptible to the onslaught of westernization?

Of all the ethnic populations studied, Asian Indian stand a very high chance of developing diabetes and cardiovascular disease at a younger age and at lower body mass indices^36^,^39^,^40^. Environmental selection of thrifty genes or thrifty phenotype and rapid westernization are potential factors responsible for the same. Recent results on diet in relation to acute myocardial infarction in the INTERHEART study puts Indians at the highest risk for susceptibility to western diet^53^. This necessitates special efforts to minimize the post-prandiol metabolic challenge of diet in relation to body size, and individual variation in response.

***The concept of small body frame:*** Salt and sugar are easily absorbed and get distributed in the total body water. Therefore, the amount consumed per drink /meal, per day needs to be matched with the body water content. Their metabolism is accompanied by a series of hormonal changes. The recommendations for reducing the sugar, salt and refined food products for children are rarely adopted in the society unlike the paediatric doses in therapeutics^18^,^54^. The sweets which are offered to children usually contain 8 to 10 times more sugar than a ordinary cup of coffee, hypothetically necessitating an intense hyperinsulinaemia to maintain euglycaemia. Such repeated and large portions of sweets consumed by children may not have any immediate clinical manifestation, but constitute a major metabolic stress to the beta cells of the pancreas. Loss of a few beta cells at that age will result in the loss of a good clone of beta cells destined to arise from them leading to the early onset of dysglycaemia in adult life^5^,^36^. We have evidence that the nephron count is lower in young individuals who are hypertensive and low birth weight children are salt sensitive^55^–^57^. But such data on the beta cells are difficult to generate, pancreas being the first organ to autolyse immediately after death. Single cola consumption is correlated with genesis of metabolic syndrome in epidemiologic studies^58^. Hence minimizing this insult will be a major dietary force to preserve the beta cells. This concept of smaller body frame is equally important in the adult life to explain the situations of obesity paradox and obese metabolically healthy individuals^59^,^60^.

***Individual variations in metabolic response to the dietary constituents:*** Indian children are predisposed to develop hyperinsulinaemia, the basic endocrine abnormality of metabolic syndrome, because of sarcopenic adiposity noted from intrauterine life^61^,^62^. Sarcopenic adiposity refers to the situation of less muscle mass and more adipose tissue for the same body mass index^60^–^63^. This is the forerunner of the premature onset of cardiovascular risk factors. This is recognized as metabolic syndrome when the risk factors cluster. Smaller body volume necessitates a hyperinsulinaemic response which is further intensified by the insulin resistance of sarcopenic adiposity. This vicious cycle of intense hyperinsulinaemia could be a logical reason for the early onset of all the risk factors at a low body mass index in Indians noted in various studies^36^–^38^.

Obesity is a major propeller of the lifestyle related disease. But in India, the thin and lean people are equally susceptible to the aftermath of high calorie western diet^36^–^38^,^40^,^41^,^64^–^68^. In other words, those of us who fail to become obese in this obesogenic environment could also end up with premature cardiovascular risk factors. The cause for sarcopenic obesity could be genetic or epigenetic, but there could be a host of modifying factors in late life like physical activity, endocrine changes, infections, drugs, stress and habituations^64^–^67^. One of the best methods to minimize the effect of sarcopenic adiposity is to improve physical activity and to reduce salt consumption^69^,^70^. In short, there could be a spectrum of body response to the metabolic stress of the food we eat. Those who have good adipocyte-beta cell response will become obese metabolically healthy individuals. Those who have a poor adipocyte and beta cell response will be lean thin and become diabetic early in life. In between, a spectrum of abnormalities could occur (Fig.) where the abnormal adipocyte response leads to hyper insulinaemia and the various risk factors now collectively termed as metabolic syndrome^71^–^75^. People in the developed countries had an opportunity to get naturally selected to become obese metabolically healthy individuals since no specific treatment was available for diabetes and metabolic syndrome when they got civilized^76^. Further, the process of westernization took around 300 years which is now abbreviated to 30 years in the developing world^77^. It is hypothesized that people in the developing countries are now paying the price for their ability to overcome this natural selection by drug treatment^68^,^76^,^77^.

**Figure 1:**
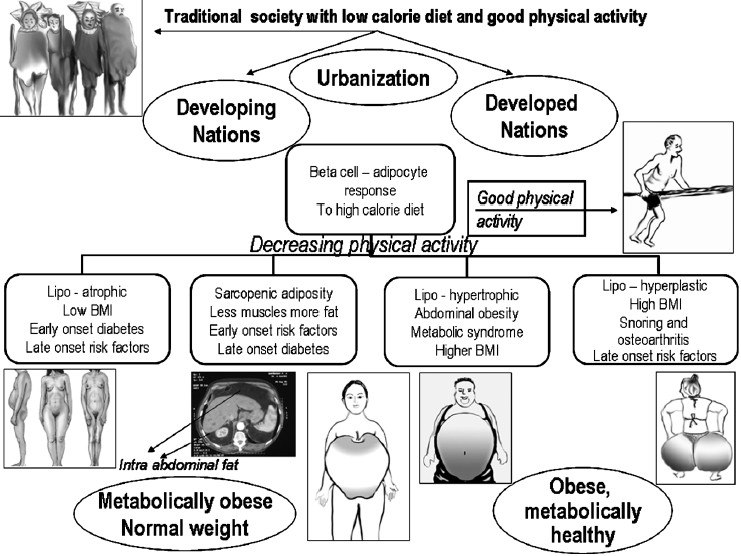
The spectrum of body response to diet and physical activity.

## The post-prandiol metabolic issues

Post-prandiol lipid abnormalities and oxidant stress are well recognized preventable components of dietary modification^78^,^79^. There is an urgent need to minimize the glycaemic, atherogenic, thrombogenic surge of every meal like what has been proposed for tobacco smoking^53^,^78^–^82^. Enteral glycaemia initiates a 3 times more intense insulin response compared to a par-enteral load mediated by the incretins^83^. In addition, there are variations in the insulin response between various races. Pima Indians have 3 times more plasma insulin levels in response to an oral glucose load compared to Europeans^77^. Recognition of the additional insult induced by the low body frame and sarcopenic adiposity add a new opportunity for dietary intervention. Individual meals need to be targeted. In addition to calorie restriction, additional efforts are needed to increase the soluble fiber content^84^. Instead of the “sumptuous thali” meal and 100 item Buffets, old way of providing minimal basal meal in restaurants with additional charge for every supplement like rice, side dishes, sweets, dessert, and curds will go a long way in minimizing the post-prandiol surge. Institution of heart healthy counters in the canteens could be a beginning where coffee and tea should be served as black coffee and weak tea with provision for adding sugar and milk only on demand. Cooked cereals (with bran) and pulses, lot of steamed vegetables, fruits, sprouted beans, fresh nuts, lean meat and steamed fish should form the menu removing the marketed and fried maladies from the canteen. The starchy fruits like banana, mango, jackfruit, roots and tubers like potato and tapioca are calorie rich and can be used as cereal substitutes only.

India has evolved as one of the top producer and consumer of the dairy products^40^. The linear relation of the growth of the dairy industry and the current cardiovascular epidemic has been analyzed for a causal relationship but not proven^40^,^85^. The ability to digest dairy product after the weaning period is an example of the environmental modification of a genetic programme. Intestinal lactase, the enzyme needed for the digestion and absorption of the milk sugar, is genetically programmed to disappear after weaning. Those of us who have lactose intolerance are those who retain this programming^86^. The metabolic impact of the dairy product is known to vary with source of the dairy product and the type of fodder provided to the herd especially with respect to polyunsaturated fats^87^. Preliminary evidence suggest that camel milk consumption may be protective against the development of diabetes^88^. Relative role of different dietary components is best illustrated by the relation of dairy products and CVD wherein dairy products are protective when the environment is more atherogenic and less protective in contrasting environs^40^,^89^.

## When to start dietary modification?

The concept of beta cell protection needs to be initiated from the time of weaning so that the biological endowment of the insulin adipogenetic system can be utilized for an extended lifetime with minimal adiposopathy^5^,^6^. The initial enthusiasm for catch up growth and overnutrition in pregnancy are now areas of further research as a harbinger for childhood onset of adult diseases^63^,^74^,^90^. This constitutes the primordial arm of dietary prevention of cardiovascular diseases. The weaning period, childhood and adolescence are equally important so that good dietary habits are cultivated and retained into adulthood. The cake provided for the birthday needs urgent replacement by a large fiber rich fruit like the watermelon since we have no control over the amount of sugar, cholesterol, trans-fats, oxidized fat, preservatives and colour the child is going to eagerly consume. More than half of the ice-creams sold in India are frozen desserts made out of harmful hydrogenated vegetable oils^50^. Some schools have already instituted healthy eating programmes by discouraging children from bringing bakery products and marketed foods for lunch and snacks.

## Oil as a cooking medium

Oil is a cooking medium for making food more palatable and to prolong shelf-life. The water content gets replaced by the oil, though the fried chips look dry^51^,^91^. The oil makes the food energy dense. The invisible fat content of the cereals and pulses contribute to 3 to 5 per cent of their weight. There is no added oil requirement for those who consume at least one non vegetarian dish a day. The invisible fat content of the Indian diet almost matches the daily requirement of 40 to 60 g per day^92^. Therefore, only vegans and those involved in heavy manual labour need added oil up to one to two table spoons if they are unable to consume the recommended daily need of 30 g of nuts. Change in dietary fats and cholesterol formed the earliest recommendation to the public which continues to be evolving^14^,^93^,^94^. In short, traditional dietary patterns which evolved with the population stand ahead in cardioprotection, among more than the 300 dietary patterns that can be evaluated^2^,^12^,^26^,^34^,^35^.

There are certain States like Kerala, in India where people are habituated to use coconut in their diet where the invisible fat content almost reaches 90 g per day^45^,^91^. Ninety per cent of the Keralites also consume at least one non-vegetarian dish a day and their calorie consumption has increased by 400 calories in the last 3 decades witnessing a high prevalence of overweight, obesity and diabetes and heart disease^45^. Fish and marine product consumption is also high in Kerala but is not credited with any beneficial effect, because of the widespread deep frying habit. Deep frying and microwave heating oxidizes the cholesterol and transforms lipid contents making them more atherogenic^95^,^96^. Polyunsaturated fats contain essential fatty acids, but have a short shelf-life, and thermal stability. It is better to use them as spreads or as seasoning. Deep frying, reheating and microwaving in addition to excessive fat consumption are dietary factors which need immediate attention. Whether there is an optimum dose for essential fatty acids, beyond which these do not have any additional protective effect, or other toxic contents like mercury in marine fish abolish the beneficial effects is currently not clear^2^,^97^.

Widely advertised omega six polyunsaturated cooking oils like sunflower oil, may not be heart friendly since the prostaglandins derived from such oils are more thrombogenic than the omega 3 polyunsaturated oils like canola, soybean and rapeseed oils^51^,^98^,^99^. Both marine fishes (consumed twice a week) as well as certain vegetable seeds like flax and fenugreek seeds can provide the daily requirement for omega 3 polyunsaturated fatty acids^98^,^100^,^101^. The recent reviews suggesting increasing the fat content of the diet to reduce the glycaemic load is likely to generate more fuel to the controversies given the role of lipids in generating metabolic syndrome^94^,^102^–^105^. The majority of vegetable oils are advertised as cholesterol free in developing countries, in an attempt to woo the consumers. In fact the sterol for the plant kingdom is ergosterol and any plant product therefore can be labelled as cholesterol free. The sterol for the animal kingdom is cholesterol and the recognition of oxidized cholesterol as an atherogenic moiety made the fats and oils of animal origin unacceptable choice as a cooking medium^51^.

## What is an ideal cardio-protective diet?

Traditional diet to which the human body has evolved and adapted forms the ideal cardio-protective diet as evidenced by the longevity and low prevalence of lifestyle related diseases in the various less civilized populations in this world^2^,^12^,^13^. The major components of this prudent diet are constituted by (a) fiber rich complex carbohydrate cereal products, (b) tree nuts and pea nuts, (c) lot of fruits and vegetables, (d) marine fish, (e) marine algae, (f) lean meat, (g) red wine, (h) and in regions away from sea shore plant products rich in unsaturated fatty acids, *e.g.*, monounsaturated fatty acids derived from olive oil and polyunsaturated fatty acids from plant sources (soy products). Thus these food components are less energy dense and have lot of flavanoids, antioxidants, vitamins and minerals. Traditionally these products are free of trans-fats, refined cereals, sugar, and excess salt. By adopting a Mediterranean type of lifestyle it is projected that 90 per cent of the type 2 diabetes, 80 per cent of the coronary artery disease, one third of the acute myocardial infarctions and 70 per cent of the strokes can be avoided^12^,^53^. There are hundreds of dietary patterns that can be adopted around the world for both primary and secondary prevention of heart disease^34^,^35^. But the low fat high carbohydrate diet is recently implicated as the reason for escalation of diabetes and heart disease since the complex carbohydrates were substituted by refined cereals and sugar^26^,^103^.

## Conclusion & future directions

Research on diet and coronary artery disease over the last one hundred years have generated more questions than what has been answered. The rapid escalation of lifestyle disease with one generation of rapid westernization in Asian Indians had opened additional avenues on research like sarcopenic adiposity and post-prandial metabolic challenges and newer modes of therapy^78^,^83^. Given the personal preferences in the dietary choices to please once own mind and taste buds, dietary guidelines are only good science which need to be modified by common sense but gets spoilt by the marketing strategies of the food industry. Ideal diet is that one which promotes health and longevity^105^. There is no simple one word solution for this complex problem where basics like meal frequency and eating behaviour need to evaluated^106^–^108^. India with its wide variation in cultural and dietary practices opens lots of avenues for research. Best example is the influence of cooking oil consumption. Currently available scientific modes of evaluation like the tissue and plasma fatty acid level estimation can act as a marker for the dietary consumption over the previous months. Such studies will be able to answer these complex issues more precisely^109^,^110^.

Rapid westernization in India has ignited a rapid escalation of lifestyle related diseases, making the country the global capital for diabetes and heart disease. The outcome of the epidemic affecting the younger age groups is devastating and is out of proportion to the epidemic of obesity. The traditional dietary advice to optimize the lipid profile, body weight, cholesterol and blood pressure has to expand to minimize the adiposopathy and loss of beta cells from the islets of pancreas from a young age. To achieve this we have to target the population at individual meals. Heart healthy counters in hotels and public functions could be the beginning. Aim is to reduce the intake of salt, sugar, cholesterol, saturated fats and trans-fats. Simultaneously we have to encourage the consumption of heart healthy components like complex carbohydrate, fruits, nuts, vegetables and fish. Healthy cooking and eating practices avoiding deep frying, needs to be popularized. Legal banning of trans-fats, salty crisps and sugar sweetened beverages is urgently needed. Appropriate food labelling of heart healthy foods will help people to make healthy choices. Substitution in the public distribution system is another choice in addition to health education and continued motivation of the public. If we cultivate a good dietary pattern in the first twenty years of our life, it will be an excellent investment for maintaining good health for the rest of our journey. For this we need a sustained revolution in the kitchen in every house and those kitchens which cater to food in the public domain by motivation if not by legislation.
